# A tribute to Ernest E. “Gene” Moore

**DOI:** 10.1186/s13017-018-0206-1

**Published:** 2018-10-04

**Authors:** Jeffry Kashuk, Walter Biffl, Yoram Kluger, Luca Ansaloni, Fausto Catena

**Affiliations:** Tel Aviv University Sackler Faculty of Medicine, Tel Aviv, Israel

Good Leaders create followers. Great leaders create leaders

-Lord Jonathan Sacks

The executive board, leadership, and membership of WSES take great pride in congratulating Professor Ernest E. “Gene” Moore on the news that the Rocky Mountain Trauma Center at Denver Health Medical Center was named the Ernest E. Moore Shock Trauma center at a ceremony in Denver, Colorado, July 10, 2018, and that a special ceremony in his honor will be held in Denver on September 29, 2018.

Prof. Moore’s ongoing legacy is an inspiration to all. His illustrious career in Trauma and Acute Care Surgery began in 1976 when he, as a young surgeon that had completed his training in Vermont under Dr. John Davis (editor, J. Trauma, 1975–1994), chose to move west and start a new life in Colorado. The young Dr. Moore quickly found himself with big shoes to fill, assuming the leadership of the Trauma services at Denver General Hospital from the illustrious Professor Ben Eiseman. Clearly, Dr. Eiseman saw in Dr. Moore a young, talented, and academically inspired young man who would carry on his trailblazing approach to academic pursuits, patient care, and advancement of the profession.

Dr. Eiseman was not to be disappointed. He lived to see Dr. Moore assumes international repute and builds the Denver Health Medical Center into one of the world’s most respected centers.

Denver Health’s unique setting where the trauma team cared for a wide variety of issues, including general, thoracic, vascular, and cardiac injuries, with the parallel emerging field of Surgical Critical Care, catapulted the Denver group into a position of being one of the founding institutions of the new emerging specialty of Acute Care Surgery. Gene had the vision and clarity to drive this agenda on the national stage, helping to create academic viability for the field and then, in later years, transforming the *Journal of Trauma* into the *Journal of Trauma and Acute Care Surgery*, highlighting the importance of the emerging specialty of Acute Care Surgery.

In the international arena, WSES takes great pride in recognizing Gene’s dedication and commitment to the establishment of our organization. In 2006, Gene joined with Fausto Catena and Luca Ansaloni to establish the *World Journal of Emergency Surgery*, for which he served as the Editor in Chief for 5 years. In 2009, the same group founded the World Society of Emergency Surgery. Gene’s dedication and commitment to our organization has helped catapult WJES into a leading journal in the field of emergency surgery, with a growing impact factor, and his suggestions and guidance have helped promote WSES as a key player in the establishment of guidelines for the field.

But Gene’s support and commitment did not end with his initial vision and support. He has remained an active participant in our organization, providing constant guidance and relevant recommendations towards the growth of our group. Simply said, when Gene rises to speak, everyone listens and takes his remarks to heart. As truly the father of our specialty, he embodies what an academic surgeon should be, combining a busy clinical practice with outstanding academic pursuits.

WSES, therefore, takes this opportunity to applaud Gene on this tremendous honor. Your colleagues, students, and friends remain eternally grateful for your contributions and look forward to your continuing contributions to our organization.

Indeed, we all deserve and look forward to MOORE!!!!!!!

With much respect, admiration, and heartfelt congratulations.

The Board and Readership of WSES and World Journal of Emergency Surgery

